# First Isolation of Hepatitis E Virus Genotype 4 in Europe through Swine Surveillance in the Netherlands and Belgium

**DOI:** 10.1371/journal.pone.0022673

**Published:** 2011-08-01

**Authors:** Renate W. Hakze-van der Honing, Els van Coillie, Adriaan F. G. Antonis, Wim H. M. van der Poel

**Affiliations:** 1 Wageningen University and Research Centre, Lelystad, The Netherlands; 2 Institute for Agricultural and Fisheries Research (ILVO), Melle, Belgium; 3 University of Liverpool, Liverpool, United Kingdom; University of Iowa, United States of America

## Abstract

Hepatitis E virus (HEV) genotypes 3 and 4 are a cause of human hepatitis and swine are considered the main reservoir. To study the HEV prevalence and characterize circulating HEV strains, fecal samples from swine in the Netherlands and Belgium were tested by RT-PCR. HEV prevalence in swine was 7–15%. The Dutch strains were characterized as genotype 3, subgroups 3a, 3c and 3f, closely related to sequences found in humans and swine earlier. The HEV strains found in Belgium belonged to genotypes 3f and 4b. The HEV genotype 4 strain was the first ever reported in swine in Europe and an experimental infection in pigs was performed to isolate the virus. The genotype 4 strain readily infected piglets and caused fever and virus shedding. Since HEV4 infections have been reported to run a more severe clinical course in humans this observation may have public health implications.

## Introduction

Hepatitis E Virus (HEV) is a small, spherical, non-enveloped single stranded, RNA virus of 27–34 nm with a genome of about 7.2 kb [1]. It is classified as a Hepevirus in the family of Hepeviridae [2]. The RNA contains a short 5′untranslated region (UTR), three overlapping open reading frames (ORF1, ORF2, and ORF3) and a short 3′UTR with a polyA-tail [3], [4]. The ORF1 encodes nonstructural proteins, ORF2 encodes the capsid protein, and ORF3 encodes a small cytoskeleton-associated phosphoprotein [5].

HEV sequences can be classified in four different genotypes, 1, 2, 3 and 4. Genotype1 and 2 circulate primarily in humans, and cause the majority of HEV infections and epidemics in Asia, Africa and Mexico [1]. The relatively conserved genotypes 1 and 2 are subclassified in five (1a–1e) and two (2a–2b) subtypes respectively [5]. Genotype 3 and 4 are primarily detected in swine, genotype 3 is mainly seen in the United States, several European countries and Japan, and genotype 4 is mainly found in Asia [5]. Genotype 3 and 4 are responsible for sporadic cases of acute Hepatitis E in humans. These genotypes are more diverse and are divided in ten (3a–3j) and seven (4a–4g) subtypes [5].

Hepatitis E virus is an important cause of acute human hepatitis in regions with inadequate water supplies and poor sanitary conditions [6]. The major transmission route of HEV infections is fecal-oral [7], usually through contaminated water. HEV causes liver inflammation, fever and jaundice in humans. The incubation period of HEV in cases where clinical symptoms arise, is 2 to 8 weeks. Chronic HEV infections have been reported but do not occur frequently [8]. In outbreaks in general the mortality is not really high (1%) [1], except in pregnant women, where this may reach 25% [9].

HEV is assumed to be a zoonotic agent: Swine have been successfully infected with human HEV [10], [11] and rhesus monkeys (experimental surrogate for human) have been infected with a swine HEV strain [12]. Eating raw swine liver and consumption of wild boar and deer has led to human cases of HEV genotype 3 [13], [14], [15]. Also, in countries previous thought to be non-endemic, 11 to 51% of subgroups like swine workers have antibodies to HEV and a general population seroprevalence of 2 to 25% has been recorded [16], [17], [18], [19], [20], [21]. Furthermore, swine origin HEVs are closely related to the human strains that were found in the sporadic human cases of acute Hepatitis E of genotypes 3 and 4 [13], [22], [23]. These observations indicate that HEV genotypes 3 and 4 infect across species, and that swine are a potential animal reservoir [24], [25], [26].

Research groups in the Netherlands have characterized different HEV strains in swine, and according to the subtyping suggested by Lu et al. 2005 [5] found subtypes 3a,3c and 3f, and since 2006 also 3e. Most strains where subtyped as 3c and 3f [27], [28], [29], [30]. In Belgium characterization of HEV strains circulating in swine was not yet reported. New HEV strains may emerge and behave different in their host. Therefore it is important to monitor the circulating strains in swine. If one particular strain becomes dominant, this may indicate that such strain is more virulent and possibly more infectious for humans. In addition, to have an insight in the potential exposure of humans to HEVs from swine we wanted to determine the actual prevalence of HEVs in swine production. To estimate approximately the percentage HEV positive pigs that could enter pork industry we tested pigs in slaughterhouses in the Netherlands and Belgium, using a real-time RT-PCR for testing of fecal samples. HEV positive samples were characterized on the ORF2 region of the virus RNA and then phylogenetically compared with known human and swine sequences.

## Materials and Methods

### Samples

Swine fecal samples in The Netherlands were collected as part of an ongoing surveillance for bacterial research. From September 16, 2008, to November 24, 2008, 101 individual fecal pig samples were collected from different pig slaughter houses (and all from different farms). All samples were collected from fattening pigs of 5 to 6 months of age, and after sampling stored for maximum one week at 4°C until testing.

Swine fecal samples in Belgium, from 23 different fattening farms were collected at a slaughterhouse; five samples from each farm, in total 115 samples. The samples were collected from fattening pigs 5 to 6 months of age. Fecal samples were resuspended in phosphate buffered saline and 10% glycerol in a (1∶ 3 dilution), and stored at −70°Cuntil testing.

### Molecular Detection of HEV

The fecal samples were mixed to a final dilution of 1∶10 in PBS. For the Dutch samples, 100 mg fecal material was mixed with 900 µl PBS and for Belgium samples 300 mg of faeces was mixed with 700 µl PBS. The 1∶10 fecal suspensions were mixed thoroughly and centrifuged in a table top centrifuge for 10 minutes at 1000× g. Two-hundred µl of supernatant was used to extract RNA with the High Pure RNA isolation kit (Roche, Mannheim, Germany). RNA was used immediately for HEV RT-PCR or stored at −70°Cuntil further testing. HEV detection by real-time RT-PCR was performed on undiluted RNA samples with the primers JVHEVF and JVHEVR as described by Jothikumar et al. [31].

### Sequencing and Phylogenetic Analysis

Fecal samples positive for HEV RNA by real-time RT-PCR were amplified using a nested RT-PCR format targeting an ORF2 fragment of HEV. Described briefly: for RT, 2 ul of 10 µM RH-HEV-Rnested primer was added to 6 µl RNA. The solution was heated for 10 minutes at 72°C and after cooling on ice, 12 µl of RTmix was added. The RT mix contained 4 µl 5× first strand buffer (Invitrogen, Breda, The Netherlands), 50 mM DTT, 0,5 mM dNTPmix(TaKaRa), 40 U RNasine (Promega, Madison, USA) and 200 U MMLV reverse transcriptase (Invitrogen, Breda, The Netherlands). The RT reaction was performed at a final volume of 20 µl. The mixture was incubated for 10 minutes at 20°C followed by 60 minutes at 42 C, heated for 5 minutes at 95°C and then placed on ice. Five µl of the RT mixture was added to the PCR mix. The first PCR was done with 15 pmol of each primer TqFwd: CTG TTY AAY CTT GCT GAC AC [32] and RH-HEV-Rnested: GAG ACA TAC ATA GGG TTG GT. The PCR mix contained 10× PCR buffer (Invitrogen, Breda, The Netherlands), 2 mM MgCl2, 0,2 mM dNTP and 2,5 U Taq DNA polymerase. The PCR reaction was performed in a final volume of 50 µl. Cycling conditions were denaturation at 95°C for 5 minutes followed by 55 amplification cycles (95°C, 1 minute, 56°C, 1 minute and 72°C, 1 minute 30 seconds), and finally heating at 72°C for 5 minutes. Five µl of the first PCR reaction is used in the nested PCR. In the nested PCR the primers ORF-s1 and ORF1-a1 were used [27], [33]. With this PCR cloned fragment of 197 nucleotides was obtained. The PCR product was separated in a 1.5% agarose gel and visualized with UV after ethidium bromide staining. Positive RT-PCR products were excised from the gel, purified by using a Gel DNA Recoverykit (Zymo Research, CA, USA), and sequenced in both directions subsequently. Sequences were submitted to Genbank.

Nucleotide sequences were aligned and clustered using Bionumerics vs 5.1 (Applied Maths) using the Jukes and Cantor correction for evolutionary rate. Evolutionary trees were drawn using Neighbor-Joining clustering. The confidence values of the internal lineages were calculated by performance of 1000 bootstrap analyses.

### Additional sequencing of genotype 4 isolate

For further characterization of the genotype 4 isolates additional sequencing was performed on isolate BeSW67HEV4-2008. On the basis of an alignment of 20 HEV genotype 4 full length sequences (data not shown) the primers HEVF1 GGCCTCACWACTGCTATTGAGC(57), HEVR1 GCRTCYTCRGARGCRfTTCCA(1129) and HEVF1nest GCCTTGGCGAATGCTGTG (105), HEVR1nest GYCTGTCCCATATATGCAGGGAC(991) were used to amplify an 887 bp fragment. For RT, 2 µl of 10 µM HEVR1 primer was added to 6 µl RNA. The solution was heated 10 minutes at 72°C and after cooling on ice, 12 µl of RTmix was added. The RT-mix contained 4 µl 5× first strand buffer (Invitrogen, Breda, The Netherlands), 50 mM DTT, 0,5 mM dNTPmix(TaKaRa), 40 U RNasine (Promega, Madison, USA) and 200 U MMLV reverse transcriptase (Invitrogen, Breda, The Netherlands). The RT reaction was performed at a final volume of 20 µl. The mixture was incubated for 10 minutes at 20°C followed by 60 minutes at 42 C, heated for 5 minutes at 95°C and then placed on ice. Five µl of the RT mixture was added to the PCR mix. The first PCR was done with 1 µM HEVF1 and HEVR1 primer, the PCR mix contains 10× pcr buffer (Invitrogen, Breda, The Netherlands), 1,5 mM MgCl2, 0,2 mM dNTP and 2,5 U Taq DNA polymerase. The PCR reaction was performed in a final volume of 50 µl. Cycling conditions were 93°C for 3 minutes and 45 amplification cycles (95°C, 30 seconds, 56°C, 30 seconds and 72°C 1 minute 30 seconds), followed by 7 minutes 72 C. Five µl of the first PCR reaction was used in the nested PCR. In the nested PCR the primers HEVFnest1 and HEVRnest1 were used, the cycling conditions were the same as the first PCR round. The PCR product was separated in a 1.5% agarose gel and visualized with UV after ethidium bromide staining. The product was excised from the gel, purified by using a Gel DNA Recoverykit (Zymo Research, CA, USA), and sequenced in both directions subsequently.

### Experimental infection in pigs

To test the infectivity of the hepatitis E virus genotype 4 isolate an experimental infection in pigs was performed. The infection experiments in pigs were approved by the ethics committee of the Animal Sciences Group, part of the Wageningen University and Research Centre, prior to the execution of the experiments (reference number 2009229/2009153.c/EXIHEV, approval date: Dec 10^th^ 2009). The review of animal experiments by this body is fully in compliance with European ethical requirements for animal experiments.

To obtain the inocula one of the HEV genotype 4 positive (RT-PCR) fecal samples from the surveillance study was diluted 1∶10 and centrifuged at 1500× g. Subsequently supernatants were filtered using a 5.0, a 0.45 and a 0.22 micropore filter, respectively. The obtained volume of 4.2 ml and was administered intravenously (Day 0) in two SPF piglets (nrs. 4392 and 4393) (2.1 ml i.v in each pig) of around 4 weeks old. All piglets used for experimental inoculation had been tested previously to be free of HEV (RT-PCR) and HEV specific antibodies (ELISA). A third piglet (4394) was not inoculated and served as a sentinel to detect HEV transmission from the inoculated animals. All three piglets were housed in one pen and were observed clinically and temperatured rectally every day. Blood samples were taken at day 0, 7, 9, 11, 14, 18, 21 and 28 post inoculation. In serum samples Alanine aminotranferase (ALT) and aspartate aminotransferase (AST) levels were analysed longitudinally by a spectrophotometric method in an automated analyser (HumaStar 89, Instruchemie, Delfzijl, The Netherlands) in all three piglets (4392, 4393 and 4394). The reference values in normal pigs used for ALT were 31–58 U/l and for AST 32–84 U/l. Fecal samples were obtained by taking rectal swabs at day 0, 7, 9, 11, 14, 16, 18, 21 and 28 post inoculation. All fecal samples were subjected to QPCR testing. In the last phase of the experiment all three piglets were euthanized to be subjected to pathological examinations and tissue sampling. The first piglet was euthanized after 2 weeks in the acute phase of the infection in order to obtain HEV positive tissue samples. The second piglet together with the sentinel piglet were euthanized and subjected to post mortem investigations 4 weeks post infection. Post mortem samples were taken of urine, feces, liver, kidney, and colon. Macroscopic pathological examinations were done in all three animals and all fecal samples, urine and liver, kidney and colon tissue samples were tested for HEV RNA by QRT-PCR (Table 1.). Excreted HEV was characterized through sequencing of PCR amplification product. All remaining samples were stored at −80°C for future studies.

**Table 1:** Hepatitis E virus shedding in experimentally infected piglets (RT-PCR Ct values in feces after inoculation, and in organs, tissues and urine at necropsy).

Day post inoculation	Piglets		
	4392†	4393†	4394‡
0	neg	neg	neg
7	neg	neg	neg
9	neg	36	neg
11	neg	32	neg
14	33	29	neg
16	30	-	neg
18	32*	-	neg
21	32	-	neg
28	neg	-	neg
liver	36/35	32/29	neg
kidney	neg	neg	neg
colum	neg	30	neg
urine	neg	neg	neg

*1∶10 dilution of the RNA, the undiluted sample showed inhibition in the PCR assay.

† intravenously inoculated piglets.

‡ Sentinel piglet.

## Results

### HEV prevalence in swine

In 15% of the101 individual Dutch fecal samples from slaughterhouses HEV was detected by real-time RT-PCR with the primer pair JVHEVF and JVHEVR. In Belgium 7% of the 115 individual samples were designated HEV positive using the same test protocol, and 5 of the 23 farms (21,7%) were designated HEV positive.

### Sequencing and Phylogenetic Analysis

All HEV- positive samples were amplified by RT-PCR targeting the ORF 2 region and amplification products were sequenced subsequently. Of 11 (of 15) and 4 (of 8) earlier positive scored samples an HEV ORF2 amplification product was obtained. The ORF2 sequences (Genbank accession HQ842716–HQ842731) were compared with published sequences of HEV genotypes from humans and swine.

**Figure 1:**
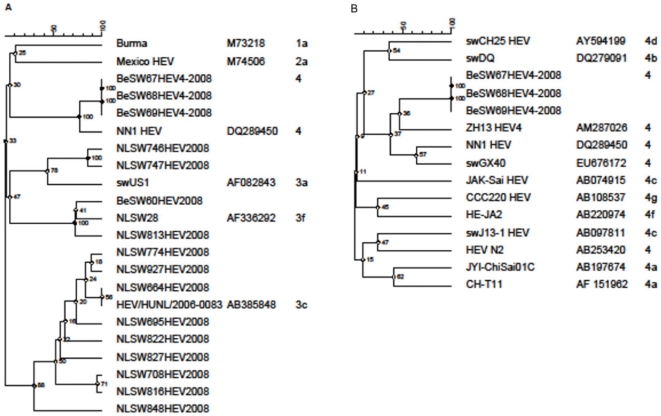
Phylogenetic tree of detected HEV sequences related to HEV sequences of humans and swine reported in literature. Analyses based on a 148 nt fragment of the ORF2 region of HEV. A) all detected HEV sequences B) genotype 4 HEV (GenBank accession HQ842716–HQ842731).

Through phylogenetic analysis using the ORF2 region, all eleven Dutch strains were characterized as genotype 3. These strains could be divided in 3 different subgroups, based on the classification scheme proposed by Lu et al [5]. Most of the strains belonged to subgroup 3c, two belonged to 3a and one belonged to 3f. The analyzed HEV strains in swine were genetically closely related to HEV isolates earlier found in humans and swine in the Netherlands (Fig. 1A). In Belgium three (BeSW67HEV4, BeSW68HEV4 and BeSW69HEV4) of the four sequences were classified as genotype 4, (seeming subtype 4b). All of these three sequences came from fecal samples from one farm. These genotype 4 sequences showed the highest similarity (97%) to strain NN1, (DQ289450), which was obtained from a Hepatitis E virus infection in rural communities in South China (Fig. 1B). The remaining Belgium sequence (BeSW60HEV4) was classified as a G3f and was closely related to (95%) NLSW28 (AF33692) (Fig. 1A).

### Additional sequencing of Genotype 4 isolate

**Figure 2:**
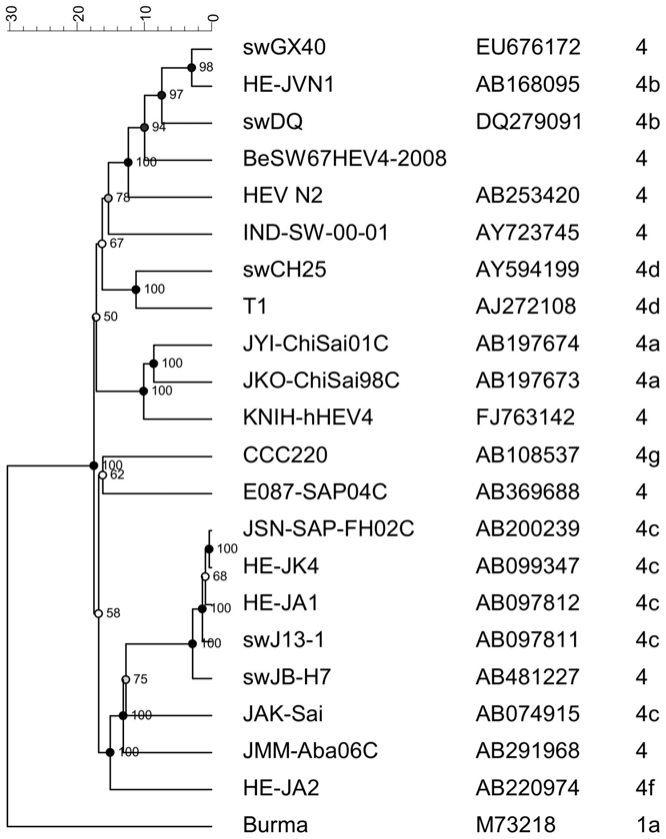
Phylogenetic tree of the HEV genotype 4 strains isolated in this study, related to HEV genotype 4 sequences of humans and swine reported in literature. Analyses based on a 887 nt fragment of the ORF1 region of HEV (GenBank accession HQ842716–HQ842731).

In addition a part of ORF1 region (bp 105–991) of the isolate BeSW67HEV4 was amplified and sequenced. The obtained sequence was compared with published sequences of HEV genotypes from humans and swine. Through phylogenetic analysis using the ORF1 region, isolate BeSW67HEV4 was characterized as a genotype 4b (Fig. 2.). The sequenced part of isolate BeSW67HEV4 was closely related to the Chinese swine strain swDQ, 90,4% similarity. The ORF2 part gave 93% similarity with strain swDQ. A comparison with the NN1 strain was not possible for this ORF1 region since the sequence thereof was not available for the NN1 strain.

### Experimental infection in pigs

**Figure 3:**
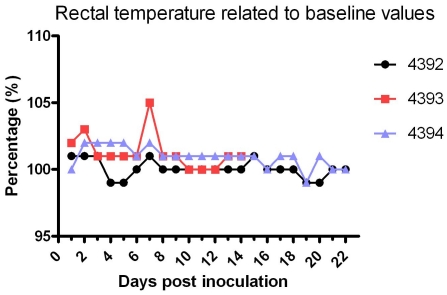
Swine body temperatures related to baseline values in experimentally (i.v) infected (nrs 4392 and 4393) and sentinel piglets (nr 4394). The average of the first 3 measurements before inoculation was taken as the baseline value.

**Figure 4:**
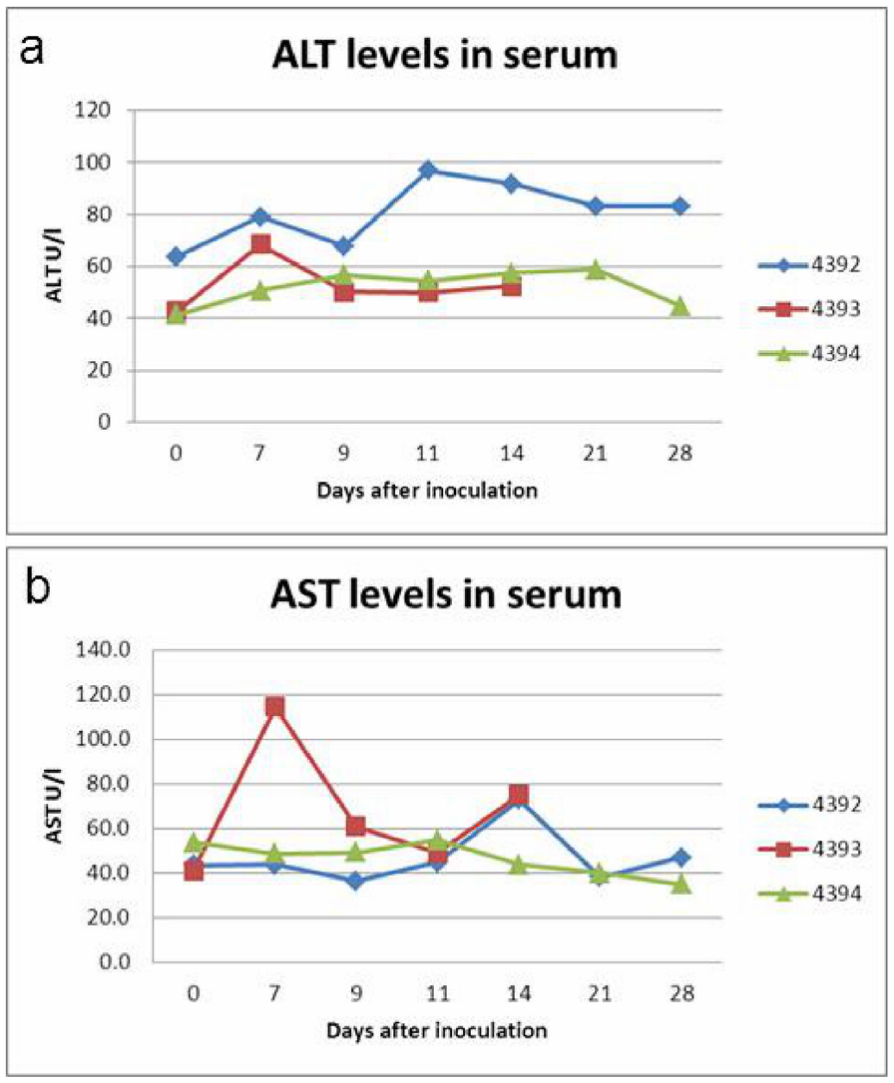
Alanine transferase (ALT) and aspartate aminotransferase (AST) values in serum. a. ALT values in serum of experimentally HEV Gt4 infected piglets (nrs. 4392 and 4393) and sentinel piglet (4394). b. AST values in serum of experimentally HEV Gt4 infected piglets (nrs. 4392 and 4393) and sentinel piglet (4394).

To test the infectivity of the hepatitis E virus genotype 4 isolate an experimental infection in pigs was performed. After inoculation, pig fecal samples were collected and tested on day 7, 9, 11, 14, 16, 18, 21 and 28 post inoculation. RT-PCR detections in fecal samples are shown in Table 1. Piglet 4393 started excreting HEV in feces at day 9 post inoculation and Piglet 4392 at day 14 (Table 1). The inoculated piglets (4392 and 4393) demonstrated virus shedding for 3 and 4 days (Table 1.), but this was not observed in the contact (sentinel) piglet. After infection piglet 4393 showed a short peak of fever at day 7 (Fig. 3.). Piglets 4392 and 4394 did not show fever although temperatures of animal 4394 (the sentinel) were relatively higher (Fig. 3.) Elevations of ALT and AST levels were observed in inoculated piglets (4392 and 4393) but not in sentinel piglets (Fig. 4.). Apart from fever no overt clinical symptoms were observed.

Hepatitis E virus genotype 4 sequences obtained from the fecal samples of the inoculated piglets were all identical to the sequence of the HEV Gt4 strain in the inoculum and the primary HEV Gt4 field sample.

## Discussion

Fecal samples of pigs in slaughterhouses in the Netherlands and Belgium, were tested for HEV using a real-time RT-PCR. HEV positive samples were characterized on the ORF2 region of the virus' RNA and phylogenetically compared with known human and swine sequences. All HEV strains detected in the Netherlands were characterized as genotype 3. All of the analyzed HEV genotype 3 strains in swine were genetically closely related to HEV isolates earlier found in humans and swine in the Netherlands. In earlier phylogenetic analyses in the Netherlands [27], [28] the most often detected genotype was 3f whereas in this study 3c was the most often detected subtype. The HEV strains found in the Belgium samples belonged to genotype 3f and genotype 4b. Since earlier information on HEV sequences from Belgium could not be retrieved comparison with such could not be made.

The detection of HEV genotype 4 in swine in Belgium was a remarkable finding. It was the first discovery of HEV genotype 4 strain in swine in Europe.

The virus readily infected swine and was isolated through swine inoculation.

In this study the virus was not naturally transmitted to a contact piglet, probably due to the short contact time.. However, it is likely that the virus will be transmitted in the field where exposures will be more frequent and last much longer. The introduction of HEV genotype 4 in swine in Europe could be an indication that genotype 4 viruses may spread over Europe and may be also to North America where genotype 3 prevalence is not much different from Europe. As a result, apart from zoonotic HEV genotype 3 in infections, we may expect zoonotic HEV genotype 4 infections in Europe as well. Since HEV4 infections have been reported to run a more severe clinical course in humans this observation may have public health implications. In this study alanine aminotranferase (ALT) and aspartate aminotransferase (AST) levels in HEV Gt4 experimentally infected piglets showed elevations which were higher than earlier reported for experimental HEV Gt3 infections [15], and may indicate more severe liver damage caused by HEV4 infections compared to HEV3 infections.

It has remained unclear how HEV genotype 4 was introduced in swine in Europe. The closest sequence was found in China but the introduction route in Europe remains unknown. To date it can only be speculated what may have been the route of introduction. Possible introduction routes include: infected animals or infected human shedding and transmitting the virus into the swine population, HEV positive animal feed or animal produce, or contaminated foods or materials, and may be other. Given the present observations of a HEV genotype 4 sequence in man in Germany [34] and the observed HEV genotype 4 sequences in swine in Belgium in this study, it can be assumed that HEV genotype 4 circulates in swine in Europe. The German human strain V0716883 [34] is closely related to the Japanese strain HE-JA3 (AB082548), genotype 4f, and there is no correlation between these genotype 4 strains. Looking at the dispersion of HEV genotype 3 in swine in Europe over the last decennium, a wider spread of HEV genotype 4 in swine in Europe may be expected. On the other hand, given the cross reactivity between HEV genotype 3 and genotype 4 strains [1], a rapid increase of HEV genotype 4 prevalence in swine in Europe may not be likely. In countries where both HEVGt3 and HEV Gt4 are detected these genotypes have been circulating together for a long time. Continued surveillance of HEV in swine will be needed to monitor changes in prevalences of the different genotypes.

This study indicates that a basic HEV surveillance in the animal reservoir can lead to the early detection of a previously undetected emerging zoonotic virus in the surveyed region. An observation that may have important public health implications.
